# Differential modulation of IL-4, IL-10, IL-17, and IFN-γ production mediated by IgG from Human T-lymphotropic virus-1 (HTLV-1) infected patients on healthy peripheral T (CD4+, CD8+, and γδ) and B cells

**DOI:** 10.3389/fmed.2023.1239706

**Published:** 2023-08-30

**Authors:** Nicolle Rakanidis Machado, Beatriz Oliveira Fagundes, Lorena Abreu Fernandes, Augusto César Penalva de Oliveira, Youko Nukui, Jorge Casseb, Fernando Roberto Machado Cunha, Luiz Henrique da Silva Nali, Sabri Saeed Sanabani, Jefferson Russo Victor

**Affiliations:** ^1^Laboratory of Medical Investigation LIM-56, Division of Dermatology, University of São Paulo, Medical School, São Paulo, Brazil; ^2^Department of Neurology, Institute of Infectology Emílio Ribas (IIER), São Paulo, Brazil; ^3^Clinics Hospital, Medical School, São Paulo, Brazil; ^4^Post Graduation Program in Health Sciences, Santo Amaro University (UNISA), São Paulo, Brazil; ^5^Laboratory of Medical Investigation LIM-03, Clinics Hospital, University of São Paulo, Medical School, São Paulo, Brazil

**Keywords:** HAM/TSP, ATLL, HTLV-1, CD4+ T, CD8+ T, IFN-γ, IL-17, IgG

## Abstract

Human T-lymphotropic virus 1 (HTLV-1) infected individuals remain as asymptomatic carriers (ACs) or can develop the chronic neurological disorder HTLV-1-associated myelopathy/Tropical Spastic Paraparesis (HAM/TSP) or the adult T-cell leukemia/lymphoma (ATLL), and the immunological mechanisms involved in this pathologies need to be elucidated. Recently, it has been demonstrated that induced or naturally developed IgG repertoires obtained from different groups of donors, grouped by immune status, can modulate human T and B cell functions. Here we aimed to evaluate if the IgG obtained from HTLV-1-infected ACs, HAM/TSP, and ATLL patients can differentially modulate the production of cytokines by human T and B cells. With this purpose, we cultured PBMCs with IgG purified from ACs, HAM/TSP, or ATLL donors and evaluated the frequency and intracellular cytokine production by flow cytometry. Our results indicate that IgG from HAM/TSP patients could induce an augment of IL-17-producing CD4+ T cells, reduce the frequency of IL-4-producing CD4+ T cells, increase IFN-γ-producing CD8+ T cells, and reduce IL-4-producing CD8+ T cells. IgG from ATLL could reduce the frequency of IL-4-producing CD4+ T cells, similarly to IgG from HAM/TSP /TSP, and could reduce the frequency of IFN-γ-producing γδT cells without influence on IL-17- and IL4-producing γδT and could reduce the frequency of IL-10- producing B cells. Finally, IgG from both HAM/TSP and ATLL patients could reduce the frequency of IFN-γ producing B cells. In conclusion, these results suggest that these preparations are active, partly overlapping in their effects, and able to elicit distinct effects on target populations.

## Introduction

HTLV-1 is the retrovirus responsible for HAM/TSP and ATLL (1–4). More frequently, HTLV-1 infections are related to a variety of inflammatory diseases, such as pulmonary alveolitis (5), uveitis (6), chronic arthropathy (7), dermatitis (8), conjunctivitis, interstitial keratitis, and others (9). Less frequently, 1 to 5% of these individuals develop ATLL or HAM/TSP, depending on mechanisms that need elucidation and are influenced by geographical location (10).

In the last years, a debate over the mechanisms mediated by differential induced or naturally developed IgG repertoires has intensified (11–13), but there is still scarce evidence to explain the molecular basis of this hypothesis termed “the hooks without bait” (14). This hypothesis suggested that the repertoire of IgG idiotypes induced by human exposure to environmental antigens, genetic background, and infections may, according to the developed repertoire, yields the generation of differential sets of IgG idiotypes that enable unforeseen interactions with clonal and conserved molecules expressed in T and B cells membranes and resulting in immune modulation. This hypothesis advocated that those IgG-lymphocyte interactions may result in regulatory or inflammatory effects with the potential to control or stimulate the developed immune response. The elucidation of this complex network may generate approaches to pathogenesis understanding and therapy development. Some studies performed under this hypothesis have reported that different IgG idiotypes repertoires can, *per se*, mediate immune modulation in murine and human lymphocytes (13, 15–19), including thymic and peripheral αβT, γδT and B cells’ cytokine production and according to the donors’ immune state. This was demonstrated when evaluating IgG from atopic individuals that modulate the production of IFN-γ by CD4+ and CD8+ T cells (20), IgG from Atopic Dermatitis patients that modulates the production of IL-17 and IL-10 by CD4+ and CD8+ T cells (21), or IgG from HIV-1-exposed non-infected and infected individuals that modulates the production of IFN-γ by αβT (CD4+ and CD8+), γδT and B cells (22).

About γδT cells, it was demonstrated that IgG from non-atopic donors could mediate the regulation of IL-17-producing γδT cells (23) and, in a similar study, also regulate IFN-γ and IL-10 production by γδT cells (24). In the context of B cells, it was recently demonstrated that human IgG from non-atopic individuals could induce IL-10-producing B cells (B10 cells) in the infant thymus and adult PBMCs (25), evidence that was generated from previous observations on murine models of allergy (26, 27). Although these studies focused on allergy development, those pieces of evidence indicate a broad spectrum of IgG-mediated regulation of cytokine production by T cells. To pave these findings from the studies cited above, its also essential to briefly highlight the following technical aspects; the described effects were obtained using as controls the commercial formulation of IgG used for human therapies, the absence of IgG, and the presence of IgG from healthy individuals; the purification method did not allow the permanence of biologically active amounts of cytokines; the IgG subclasses on all formulations were similar; the IgG formulations could directly interact with lymphocytes membranes that do not express IgG receptors; the IgG-membrane interactions did not result in the induction of apoptosis.

In the context of HTLV-1 infected patients, it was demonstrated that, compared to healthy donors or asymptomatic carriers, the HAM/TSP patients are characterized by elevated levels of pro-inflammatory cytokines, such as IL-4, IL-6, IL-8, IFN-γ, and Tumor Necrosis Factor-α (TNF-α) in their plasma (28, 29). It was also demonstrated that increased plasma levels of IL-17 can be detected in HAM/TSP patients (30), and the plasma levels of IL-10 may be elevated in some subtypes of ATLL patients (31). Using a murine model of ATLL, it was also demonstrated that IL-10 production may be significant in defining the ATLL progression (32).

The control of HTLV-1 proliferation in infected individuals is dependent on the induction of effector immune mechanisms, including the production of IgG antibodies that recognize the virus and stimulate neutrophil-mediated cytotoxic responses to HTLV-1-infected cells (27). Immunological stimulation in the decurrence of HTLV-1/host interaction may influence the clinical evolution of lifelong asymptomatic HAM/TSP or ATLL patients. Furthermore, it was described an association between HAM/TSP development with high levels of HTLV-1 antibodies (33), indicating that the intensity and/or specificity of the humoral response may, at some point, be related to the progression of the HTLV-1 infection and the manifestations of inflammatory associated diseases. Moreover, the intense inflammatory activity due to the CD4+ T cells activation, with increased levels of IFN-γ, contributes to the establishment of the chronic inflammatory process (34).

Based on those observations, in the present study, we aimed to evaluate the effect of the IgG repertoires obtained from asymptomatic, HAM/TSP, and ATLL donors on the production of cytokines by peripheral αβT- (CD4+ and CD8+), γδT- and B-cells searching for the identification of differential immunological signatures that may be related to each clinical manifestations resultant from HTLV-1 infection.

## Methods

### Samples

Purified IgG was obtained from randomly selected patients from a larger cohort of 233 HTLV-1-infected persons representing asymptomatic carriers (ACs; *n* = 14; 12 males and 2 females; Age: 52.7 ± 2.7; Pro-viral load median: 13 copies/1000 PBMCs), HAM/TSP (*n* = 16; 10 males and 6 females; Age: 57.4 ± 2.2; Pro-viral load median: 162 copies/1000 PBMCs), and ATLL (*n* = 11; 7 males and 4 females; Age: 48.4 ± 4.6; Pro-viral load median: 502 copies/1000 PBMCs) patients. HTLV-1-positive individuals were recruited from the HTLV-1 outpatient clinic at the University of São Paulo and the Institute of Infectious Diseases “Emilio Ribas.” All ACs were diagnosed as HTLV-1 carriers at the time of blood donation. Viral infection was identified by the Murex HTLV I + II (Abbott/Murex, Wiesbaden, Germany) and Vironostika HTLVI/II (bioMérieux bv, Boxtel, Netherlands) HTLV enzyme immunoassays, and infection was confirmed by HTLV BLOT 2.4 (HTLV blot 2.4, Genelabs Diagnostics, Science Park, Singapore). The clinical status of HAM/TSP was determined based on the WHO criteria for HTLV-1-associated diseases (35). Diagnostic criteria for ATLL included serologic evidence of HTLV-1 infection and cytologically or histologically proven T cell malignancy. Healthy controls (HCs) were volunteers that were diagnosed as non-infected individuals at the time of blood donation. Blood samples from healthy controls were used to obtain purified IgG (*n* = 30) or to obtain the PBMCs (*n* = 10) used in the culture experiments avoiding autologous IgG-PBMCs experiments. Written informed consent was obtained from each participant. The study was approved by the local review board.

### IgG purification

IgG was purified from pooled serum following the Melon Gel IgG Spin Purification Kit protocol (Thermo, Waltham, MA, United States). Purified IgG was collected, sterilized using 0.20-micron filters (Corning, Darmstadt, Germany), and stored at −80°C for cell culture experiments. Following the manufacturer’s instructions, IgG concentrations were determined using Coomassie Protein Assay Reagent (Pierce, Waltham, MA, United States). The purity of IgG, as evaluated by SDS-PAGE, was greater than 95%, and the 5% contaminants had molecular weight lower than 10 kDa indicating that they are predominantly protein fragments that the melon gel could not capture and that they probably do not include molecules of biological importance such as cytokines. All pools were evaluated, and undetectable IgA, IgM, and IgE antibody levels were confirmed. The frequency of IgG subclasses, as evaluated by ELISA, was similar between all IgG formulations. This purification protocol was evaluated for the quality of the purified IgG, and it demonstrated higher efficiency in purifying idiotype-functional molecules and avoiding complex IgG molecules (36). Furthermore, the serum samples were exposed to UV radiation and long-period freezing to avoid the presence of infective HTLV-I.

### Cell culture with PBMCs from healthy donors

Suspensions of PBMCs from healthy individuals were washed and resuspended in RPMI 1640 medium containing 10% FC-III (HyClone, Logan, UT, United States). Using a Neubauer chamber, a cell suspension aliquot was diluted in trypan blue (Sigma, United States) to evaluate the cell viability and number. Then, 1 × 10^6^ viable PBMCs were placed in each well of a 96-well culture plate (Costar, Glendale, AZ, USA) and cultured with 100 μg/mL IgG purified from pooled serum samples from each group of patients or controls in RPMI 1640 medium containing 10% FC-III (HyClone, Logan, UT, USA). The mock condition or the addition of 100 μg/mL commercially purified IgG (IVIg) was used as an additional control. The culture plates were incubated for 3 days, and 1 μg/mL brefeldin A (Sigma, Israel) was added in the last 12 h as previously standardized (20, 21, 23, 24, 37). Cell staining was performed to evaluate cell labeling via flow cytometry.

### Flow cytometry

Cell culture and flow cytometry were performed using protocols that were previously described by our group (20, 21, 38, 39). PBMCs were transferred to test tubes to perform extracellular staining, and 1 μg of each antibody was added to the cells (except to the unlabelled tubes). Then, the samples were incubated for 30 min at four °C while protected from light. Thereafter, 500 μL of PBS solution was added, and the tubes were centrifuged. The supernatant was discarded by inverting each tube. Then, PBS was added, followed by fixation in 200 μL of 1% formaldehyde for at least 10 min. PBMCs were stained with mouse anti-human CD3, γδTCR, CD4, CD8, CD19, or isotype control antibodies (BD Pharmingen, NJ, United States). The tubes were centrifuged, the supernatant was discarded, and the ideal concentration for each antibody (determined by previous titration experiments) was added to the cells (except to the unlabelled tubes) to perform intracellular labeling. Then, 100 μL of PBS containing 0.05% saponin was added, and the tubes were stored at 4°C for 30 min while protected from light. After centrifugation, the supernatant was discarded by inverting each tube, and the cells were resuspended in 300 μL of PBS solution. PBMCs were stained with mouse anti-human IFN-γ, IL-14, IL-10, and IL-17 or isotype control conjugated with the corresponding fluorochromes (BD Pharmingen, New Jersey, United States). Using an LSRII Fortessa flow cytometer (BD Biosciences, United States), 500,000 events per sample were acquired in the quadrant of lymphocytes (as determined by their relative size/granularity). Compensation was performed using adsorbed microspheres (CompBeads, BD Biosciences, USA) treated with the antibodies used for extra- and intracellular staining. Cell gating was based on the specific isotype control to identify T and B cells gating, and all the cytokines gating was verified using the fluorochrome minus 1 (FMO) setting where all the antibodies needed to perform the phenotypic labeling were added except for the one needed to label each of the cytokines. CD3 + γδTCR+ lymphocytes were considered γδT cells, CD8-CD4-CD19+ lymphocytes were considered B cells, CD4 + CD8- lymphocytes were considered CD4+ T cells, and CD8 + CD4- lymphocytes were considered CD8+ T cells. For the cell viability analysis, the cells were incubated with Live/Dead (PE-Texas red) fluorescent reagent (ThermoFisher, United States), and all analyses were performed using viable cells. Data analysis was performed using FlowJo software (Tree Star, Ashland, OR, United States).

### Statistical analysis

Statistical analysis was performed with GraphPad Prism 5.0 (GraphPad Software Inc., La Jolla, CA). Data from *in vitro* studies were taken from 5 separate experiments with 2 PBMC donors per experiment. Differences were considered significant at *p* ≤ 0.05 as assessed by Student’s *t*-test and the Mann–Whitney U test (comparisons between two groups) or by an ANOVA test with Tukey’s test correction (comparisons among more than two conditions) because of the Gaussian distribution of the values.

## Results

### Differential effects of IgG from ACs, HAM/TSP, and ATLL patients on the IL-4, IL-10, IL-17, and IFN-γ production by T (CD4+, CD8+, and γδ) and B cells

To evaluate the effects of grouped IgG idiotypes repertoires, we purified and pooled IgG antibodies from ACs, HAM/TSP, and ATLL patients and performed culture experiments using PBMC from healthy non-infected individuals. As control conditions, all experiments included the mock condition (absence of IgG), the IVIg condition (therapeutical IgG formulation), and the HCs condition (IgG from healthy non-infected donors who did not donate PBMC).

**Figure 1:**
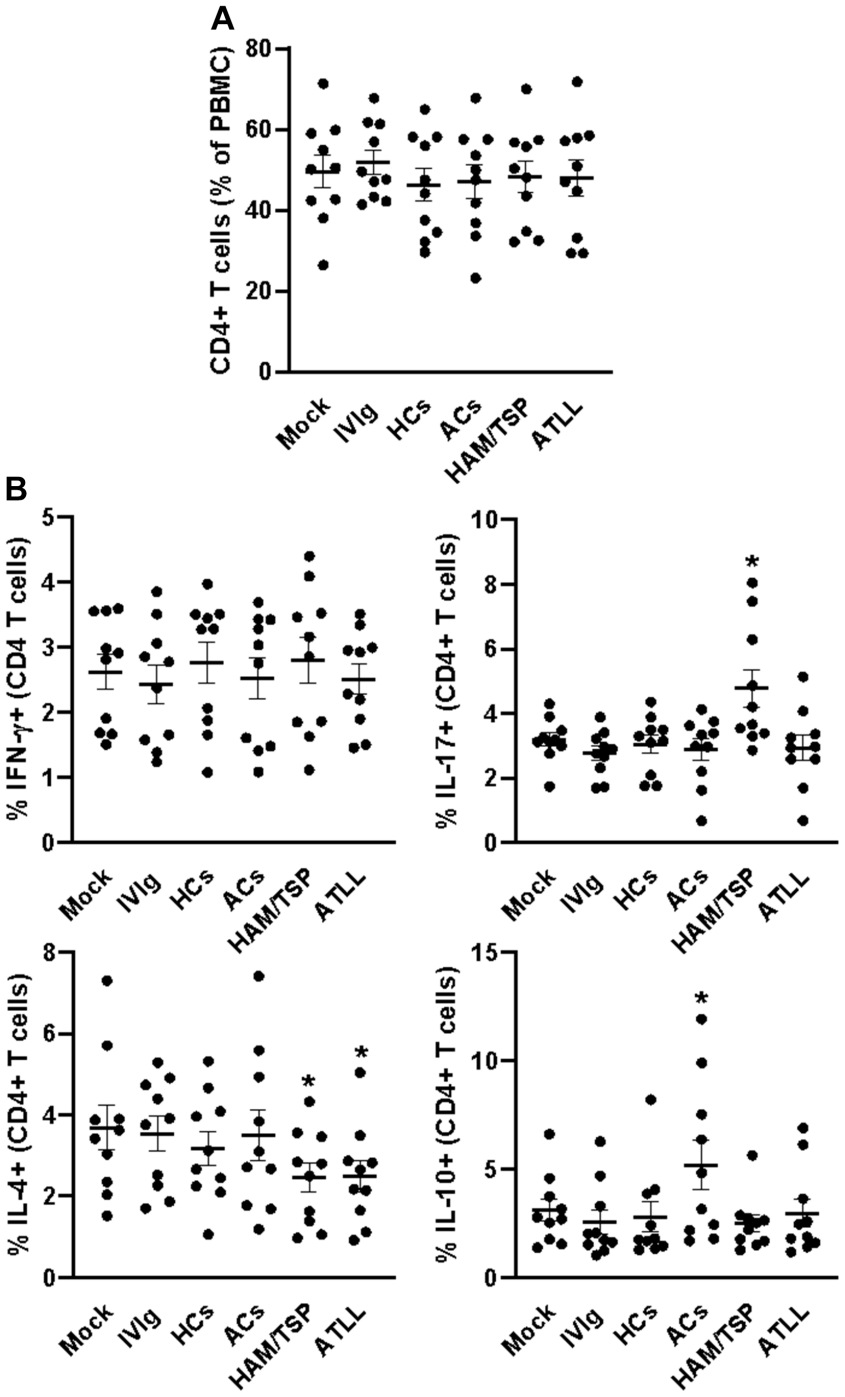
Modulatory effect of IgG from HTLV-1-infected patients on healthy peripheral CD4+ T cells. PBMCs from healthy donors (*n* = 10) were cultured in the absence of IgG (Mock) or in the presence of 100 μg/mL of IgG used for therapeutical proposes (IVIg), IgG purified from healthy non-infected controls (HCs), IgG from HTLV-1-infected asymptomatic carriers (ACs), IgG from HTLV-1-infected HAM/TSP P/TSP patients (HAM/TSP P/TSP) or IgG from HTLV-1-infected ATLL patients. After 3 days of culture, viable CD4+ T cells were evaluated by flow cytometry for frequency **(A)** and intracellular production of IFN-γ, IL-17, IL-4, and IL-10 **(B)**. Symbols represent individual values obtained from five experiments, and lines represent mean ± SE. ^*^ = *p* < 0.05 compared to the Mock, IVIg, and HCs conditions.

**Figure 2:**
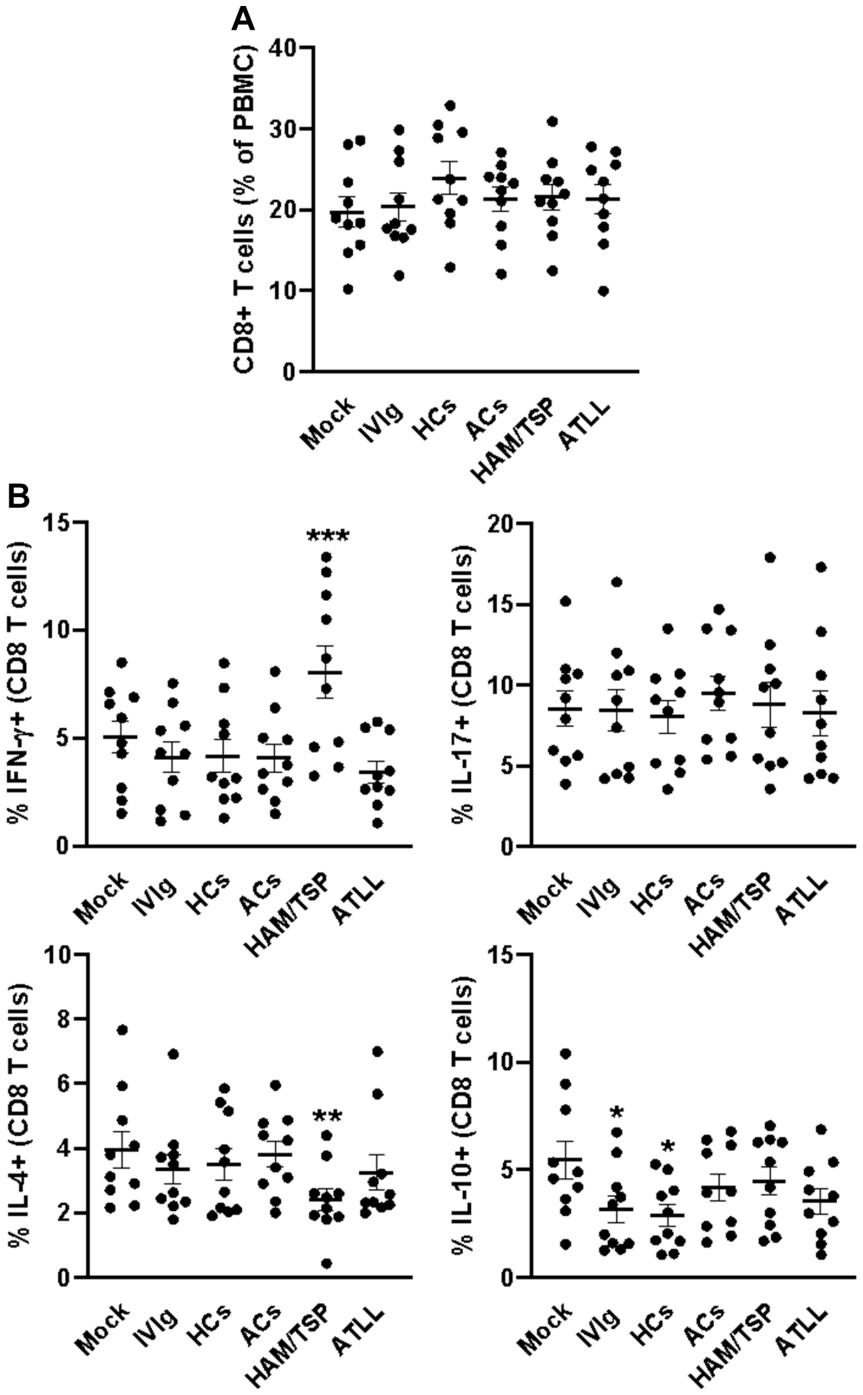
Modulatory effect of IgG from HTLV-1-infected patients on healthy peripheral CD8+ T cells. PBMCs from healthy donors (*n* = 10) were cultured in the absence of IgG (Mock) or in the presence of 100 μg/mL of IgG used for therapeutical proposes (IVIg), IgG purified from healthy non-infected controls (HCs), IgG from HTLV-1-infected asymptomatic carriers (ACs), IgG from HTLV-1-infected HAM/TSP P/TSP patients (HAM/TSP P/TSP) or IgG from HTLV-1-infected ATLL patients. After 3 days of culture, viable CD8+ T cells were evaluated by flow cytometry for frequency **(A)** and intracellular production of IFN-γ, IL-17, IL-4, and IL-10 **(B)**. Symbols represent individual values obtained from five experiments, and lines represent mean ± SE. ^***^ = *p* < 0.05 compared to all other conditions. ^**^ = *p* < 0.05 compared to the Mock, IVIg, and HCs conditions. ^*^ = *p* < 0.05 compared to the Mock condition.

After 3 days of culture, all evaluated culture conditions did not influence the frequency of CD4+ T cells (Figure 1A and Supplementary Figure S1A), and a similar equality profile was observed in the IFN-γ-producing CD4+ T cells (Figure 1B and Supplementary Figure S2). When evaluating the IL-17 production, we could observe that IgG from HAM/TSP patients could induce an augment of IL-17-producing CD4+ T cells compared to all other conditions (Figure 1B and Supplementary Figure S2). IL-4 analyses could reveal that IgG from HAM/TSP and ATLL could reduce the frequency of IL-4-producing CD4+ T cells compared to other conditions, and the evaluation of IL-10 production indicates that IgG from ACs induces the production of IL-10 by CD4+ T cells compared to all other conditions (Figure 1B and Supplementary Figure S2). We next evaluated the frequency of CD8+ T cells, and we could observe no influence on the frequency of this population between all culture conditions (Figure 2A and Supplementary Figure S1A). Otherwise, IgG from HAM/TSP patients could induce an augment of IFN-γ-producing CD8+ T cells and a reduction of IL-4-producing CD8+ T cells compared to other culture conditions (Figure 2B and Supplementary Figure S3). No difference was observed in the production of IL-17 by CD8+ T cells between all culture conditions. Evaluating IL-10 production, we could observe that IVIg and IgG from HCs could reduce the frequency of IL-10-producing CD8+ T cells compared to the mock condition, an effect that was not observed in response to IgG from ACs, HAM, and ATLL (Figure 2B and Supplementary Figure S3).

**Figure 3:**
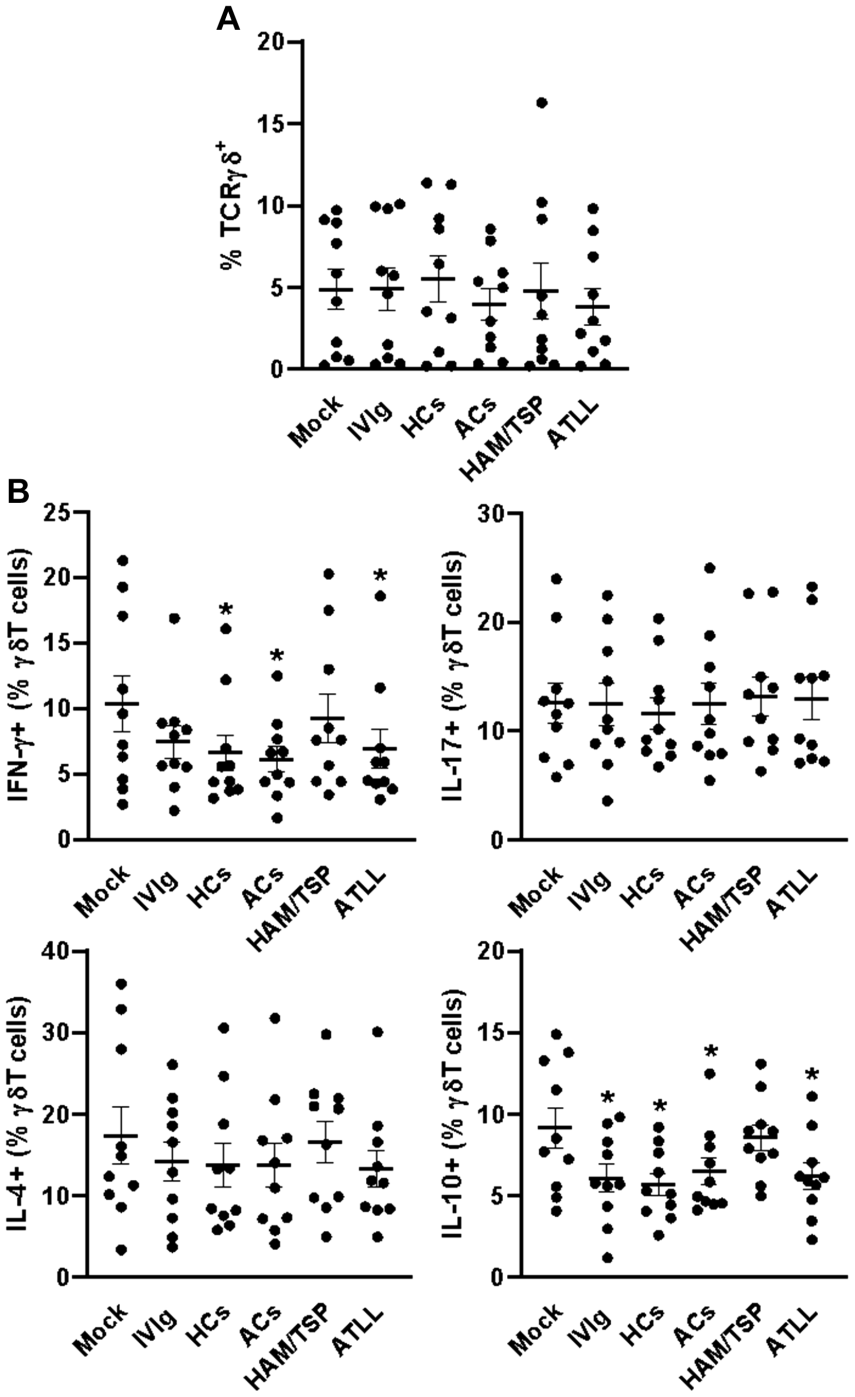
Modulatory effect of IgG from HTLV-1-infected patients on healthy peripheral γδT cells. PBMCs from healthy donors (n = 10) were cultured in the absence of IgG (Mock) or in the presence of 100 μg/mL of IgG used for therapeutical proposes (IVIg), IgG purified from healthy non-infected controls (HCs), IgG from HTLV-1-infected asymptomatic carriers (ACs), IgG from HTLV-1-infected HAM/TSP P/TSP patients (HAM/TSP P/TSP) or IgG from HTLV-1-infected ATLL patients. After 3 days of culture, viable γδT cells were evaluated by flow cytometry for frequency **(A)** and intracellular production of IFN-γ, IL-17, IL-4, and IL-10 **(B)**. Symbols represent individual values obtained from five experiments, and lines represent mean ± SE. ^*^ = *p* < 0.05 compared to the Mock condition.

When evaluating γδT cells, we could not detect any differences in the frequency of these cells between culture conditions (Figure 3A and Supplementary Figure S1B). Otherwise, IgG from HCs, ACs, and ATLL patients could reduce the frequency of IFN-γ-producing γδT cells compared to the mock condition. No influence of any condition could be detected in the IL-17- and IL4-producing γδT cells between all culture conditions. The production of IL-10 could be detected at a lower frequency in response to IVIg and IgG from HCs, ACs, and ATLL patients compared to mock condition (Figure 3B and Supplementary Figure S4).

**Figure 4:**
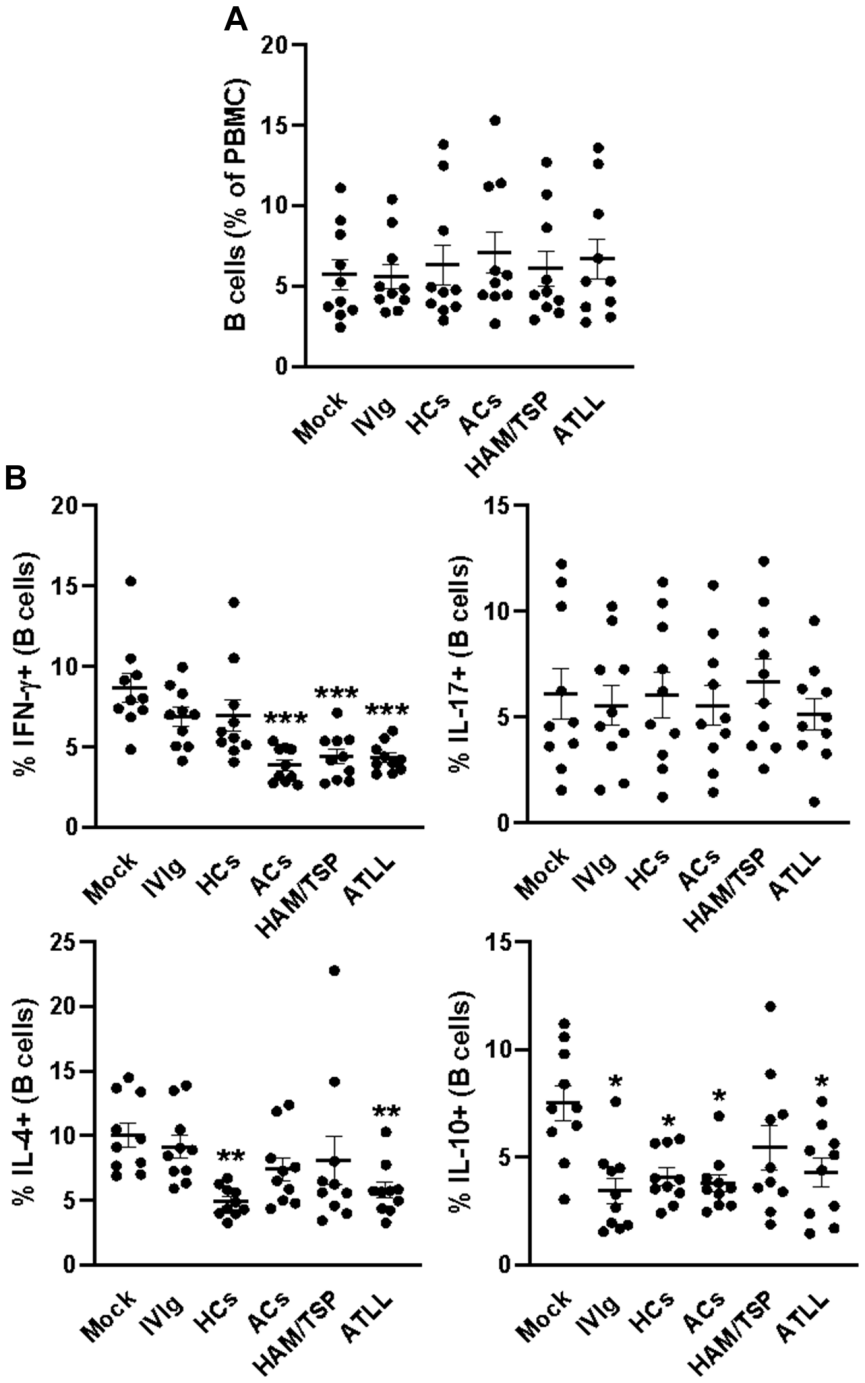
Modulatory effect of IgG from HTLV-1-infected patients on healthy peripheral B cells. PBMCs from healthy donors (*n* = 10) were cultured in the absence of IgG (Mock) or in the presence of 100 μg/mL of IgG used for therapeutical proposes (IVIg), IgG purified from healthy non-infected controls (HCs), IgG from HTLV-1-infected asymptomatic carriers (ACs), IgG from HTLV-1-infected HAM/TSP P/TSP patients (HAM/TSP P/TSP) or IgG from HTLV-1-infected ATLL patients. After 3 days of culture, viable CD8+ T cells were evaluated by flow cytometry for frequency **(A)** and intracellular production of IFN-γ, IL-17, IL-4, and IL-10 **(B)**. Symbols represent individual values obtained from five experiments, and lines represent mean ± SE. ^***^ = p < 0.05 compared to the Mock, IVIg, and HCs conditions. ^**^ = *p* < 0.05 compared to the Mock and IVIg conditions. ^*^ = *p* < 0.05 compared to the Mock condition.

Ultimately, we also evaluated the frequency of B cells after the culture period, and we could not detect any differences in the frequency of these cells between culture conditions (Figure 4A and Supplementary Figure S1A). By evaluating the IFN-γ production, we could observe that IgG from ACs, HAM, and ATLL patients could reduce the frequency of IFN-γ-producing B cells compared to mock, IVIg, and HCs controls (Figure 4B and Supplementary Figure S5). No influence was observed when comparing the production of IL-17 by B cells between all culture conditions. The frequency of IL-4-producing B cells was reduced in response to IgG from HCs and ATLL patients compared to mock and IVIg conditions (Figure 4B and Supplementary Figure S5). By evaluating the production of IL-10, we could also observe that IVIg, IgG from HCs, ACs, and ATLL patients could reduce the frequency of IL-10-producing B cells compared to mock conditions (Figure 4B and Supplementary Figure S5).

## Discussion

HAM/TSP is potentially considered an immune-mediated disease, mainly due to impaired production of cytokines, including IFN-γ levels (36). Our observations suggested that IgG molecules may influence the described impairment and may be divergent between peripheral lymphocyte populations. Our study indicated that IgG from HAM/TSP patients could induce a remarkable augment on the production of IFN-γ by CD8+ T cells without influence on the production by CD4+ T cells and γδT cells and with a reduction on B cells. Furthermore, this observation corroborates the description that a subset of IFN-stimulated genes is overexpressed in HAM/TSP cases but not asymptomatic carriers (40). Moreover, it was demonstrated that the infiltration of HTLV-1-specific CD8+ T cells is related to HAM/TSP development (41). This effect seems to occur as a bystander neural damage mediated by the apoptosis of oligodendrocytes that were in contact with infiltrated CD8+ T cells against HTLV-1-infected CD4+ T cells. Again, our observation that only IgG from HAM/TSP patients could induce the augment of IFN-γ in CD8+ T cells corroborates with the suggested mechanism for disease development. It is interesting to highlight that it was described an association between the IFN-γ gene polymorphism and the plasma levels of this cytokine in HTLV-1-infected individuals indicating an IFN-γ-mediated response in a shorter timeframe as a trigger to HTLV-1-related symptoms (42). Transposing our findings to *in vivo* conditions, the IgG antibodies may collaborate, triggering an IFN-γ-mediated response and HTLV-1-related symptoms and favoring HAM/TSP development, an aspect that needs further elucidation.

Of note, our results also indicated that IgG from HAM/TSP patients could induce a higher frequency of IL-17-producing CD4+ T cells (Th17) without influencing the production of this cytokine in all other evaluated populations. This evidence corroborates with the literature that demonstrated increased plasma levels of IL-17 in HAM/TSP patients (30) since Th17 cells are the major IL-17 producers in peripheral blood. Additionally, it was demonstrated that the IL-17 production observed in HAM/TSP patients is not directly associated with the genetic background of the infected individuals since it was described the lack of association between the polymorphism in the IL-17 gene and the development of HAM/TSP (43). This evidence corroborates with a possible role of IgG in regulating the production of IL-17 against HTLV-1 infection and modulating HAM/TSP development. However, it is essential to highlight that the pivotal role of Th17 cells demonstrated in another central nervous system disease, multiple sclerosis, is not established in HAM/TSP (44), demanding more investigations on this topic.

When evaluating the production of IL-4 in response to pooled IgG, we could observe that IgG from ATLL and HAM/TSP patients inhibits the production of IL-4 by its primary source, the CD4+ T cells. This observation was significant since it was described that IL-4 could induce leukemic cell proliferation *in vitro* (45, 46) and that ATLL patients have a high expression of the IL-4 receptor (IL-4R) on the surface of leukemic cells from acute ATLL patients (47). Linking those pieces of evidence to our findings, the IgG repertoire induced in ATLL patients may play a protective role by mediating a negative regulation of IL-4 production that may negatively impact the proliferation of leukemic cells. Otherwise, some studies that evaluated the peripheral levels of IL-4 in HTLV-1-infected individuals did not demonstrate an association with HAM/TSP development (30, 48), suggesting that the observed IgG effect on IL-4 production may not play a substantial role in HAM/TSP pathogenesis.

We also evaluated the production of IL-10, and we could observe that IgG from asymptomatic carriers could induce augmented levels of IL-10-producing CD4+ T cells. The high levels of CD4+ T cells producing IL-10 were described in asymptomatic carriers as a possible immunoregulatory mechanism that guarantees their asymptomatic clinical status (49), and IL-10 blockage in ATLL patients collaborates with leukemia-initiating cell eradication (32). It was also demonstrated that HAM/TSP patients treated with pentoxifylline upregulate the production of IL-10, which may account for clinical improvement (50). Therefore, the IL-10 production induced in CD4+ T cells by IgG from asymptomatic carriers may collaborate with a clinical control of the disease or infection control on patients submitted to therapeutic protocols. This observation is also essential because IL-10 production is unrelated to the genetic polymorph (50), increasing the possible importance of controlling IL-10 production mediated by IgG antibodies.

Since 1990, much has been discussed about the relationship between genotypes and HTLV-1 disease progression (51). Until recent years many polymorphisms had been described as related to the progression of HTLV-1infection including human leucocyte antigen (HLA) genes (52), the immunoregulation-related gene FOXP3 (53), the vascular endothelial growth factor (VEGF) gene (54), Tax-responsive elements (TRE) gene (55), Mannose-binding lectin-associated serine protease 2 (MASP2) gene (56) and others that could be related to the pro-viral load control or development of clinical manifestations including HAM/TSP. In our *in vitro* model, the ACs, HAM/TSP, and ATLL patients donated only IgG antibodies, indicating that genetic factors related to any molecules other than the genes related to donnor’s IgG production could not influence the observed results. Furthermore, all PBMC samples used in our study were submitted to all culture conditions mitigating the genetic variations background between conditions and at the cellular level.

The culture protocol used in the present study allows the evaluation of the IgG repertoire effect without multiple genetic influences but had a significant limitation because we did not evaluate individual genetic aspects of the PBMC donors (ethnic origin and others), which we intend to evaluate in future studies. Although, our results indicate that the IgG repertoire may, at some point, influence the cellular immune response profile in biomarkers that can influence HTLV-I pathogenesis. This aspect was scarcely discussed in the literature, but it was demonstrated 20 years ago that IgG isolated from HAM/TSP patients could recognize the human heterogeneous nuclear ribonuclear protein-A1 (hnRNP-A1). This molecule could be listed as a target molecule mediating HAM/TSP development (57). In the same year, it was proposed that HTLV-I-specific IgG crossreaction with human conserved molecules could represent a pathological mechanism for HAM/TSP induction in HTLV-I-infected individuals (58). Unfortunately, after this suggestion, we did not find studies that could relate the production of IgG antibodies and the pathogenesis of individuals infected by HTLV-I, to the point that we have found a recent review article that no longer mention the production of antibodies as a participant in the development of HAM/TSP (59).

In conclusion, our observations could demonstrate that different populations of peripheral T (CD4+, CD8+, and γδ) and B cells can differentially produce IFN-γ, IL-4 IL-10, and IL-17 according to the repertoire of IgG produced by each group of HTLV-1-infected individuals (AC, HAM, and ATLL), what may collaborate on framing the control or development of disease. These observations open a new field of investigation that may collaborate with elucidating HTLV-1 infection pathogenesis.

## Data availability statement

The raw data supporting the conclusions of this article will be made available by the authors, without undue reservation.

## Ethics statement

The studies involving humans were approved by The study was approved by the review board of the University of São Paulo, Medical School, São Paulo, Brazil. The studies were conducted in accordance with the local legislation and institutional requirements. The participants provided their written informed consent to participate in this study.

## Author contributions

NM, BF, and LF performed *in vitro* experiments and data analysis. AO, YN, and JC carried out the diagnosis and selection of patients. FC performed *in vitro* experiments. LN and SS critically revised the manuscript. JV coordinated the activities of all other collaborators, obtained funding to carry out the study and wrote the manuscript. All authors contributed to the article and approved the submitted version.

## Funding

This study was funded by grants from the Laboratory of Medical Investigation-56, Medical School, University of São Paulo, São Paulo, Brazil (LIM-56 HC-FMUSP), National Council for Scientific and Technological Development (CNPq) grant# 302937/2021-8 and São Paulo Research Foundation (FAPESP) grant #2021/08225-8.

## Conflict of interest

The authors declare that the research was conducted in the absence of any commercial or financial relationships that could be construed as a potential conflict of interest.

## Publisher’s note

All claims expressed in this article are solely those of the authors and do not necessarily represent those of their affiliated organizations, or those of the publisher, the editors and the reviewers. Any product that may be evaluated in this article, or claim that may be made by its manufacturer, is not guaranteed or endorsed by the publisher.

## References

[1] Gessain A Barin F Vernant JC Gout O Maurs L Calender A. Antibodies to human T-lymphotropic virus type-I in patients with tropical spastic paraparesis. Lancet. (1985) 2:407–10. doi: 10.1016/s0140-6736(85)92734-5, PMID: 2863442

[2] Osame M Usuku K Izumo S Ijichi N Amitani H Igata A. HTLV-I associated myelopathy, a new clinical entity. Lancet. (1986) 1:1031–2. doi: 10.1016/s0140-6736(86)91298-5, PMID: 2871307

[3] Poiesz BJ Ruscetti FW Gazdar AF Bunn PA Minna JD Gallo RC. Detection and isolation of type C retrovirus particles from fresh and cultured lymphocytes of a patient with cutaneous T-cell lymphoma. Proc Natl Acad Sci U S A. (1980) 77:7415–9. doi: 10.1073/pnas.77.12.7415, PMID: 6261256 PMC350514

[4] Yoshida M Miyoshi I Hinuma Y. Isolation and characterization of retrovirus from cell lines of human adult T-cell leukemia and its implication in the disease. Proc Natl Acad Sci U S A. (1982) 79:2031–5. doi: 10.1073/pnas.79.6.2031, PMID: 6979048 PMC346116

[5] Sugimoto M Nakashima H Watanabe S Uyama E Tanaka F Ando M. T-lymphocyte alveolitis in HTLV-I-associated myelopathy. Lancet. (1987) 2:1220. doi: 10.1016/s0140-6736(87)91362-6 2890850

[6] Huang Y. Q. Li J. J. Nicolaides A. Zhang W. G. Freidman-Kien A. E.: Fibroblast growth factor 6 gene expression in AIDS-associated Kaposi's sarcoma. Lancet. (1992) 339:1110–1. doi: 10.1016/0140-6736(92)90702-5, PMID: 1349122

[7] Nishioka K Maruyama I Sato K Kitajima I Nakajima Y Osame M. Chronic inflammatory arthropathy associated with HTLV-I. Lancet. (1989) 1:441. doi: 10.1016/s0140-6736(89)90038-x, PMID: 2563817

[8] Batista ES Oliveira PD Primo J Varandas CMN Nunes AP Bittencourt AL. HTLV-1 proviral load in infective dermatitis associated with HTLV-1 does not increase after the development of HTLV-1-associated myelopathy/tropical spastic paraparesis and does not decrease after IDH remission. PLoS Negl Trop Dis. (2019) 13:e0007705. doi: 10.1371/journal.pntd.0007705, PMID: 31851683 PMC6946163

[9] Martin F Taylor GP Jacobson S. Inflammatory manifestations of HTLV-1 and their therapeutic options. Expert Rev Clin Immunol. (2014) 10:1531–46. doi: 10.1586/1744666X.2014.966690, PMID: 25340428

[10] Gessain A Cassar O. Epidemiological aspects and world distribution of HTLV-1 infection. Front Microbiol. (2012) 3:388. doi: 10.3389/fmicb.2012.00388, PMID: 23162541 PMC3498738

[11] de Sousa TR Victor JR. Natural self-ligand Gamma Delta T cell receptors (γδTCRs) insight: the potential of induced IgG. Vaccines (Basel). (2020) 8:436. doi: 10.3390/vaccines8030436, PMID: 32759782 PMC7564284

[12] Victor JR. Allergen-specific IgG as a mediator of allergy inhibition: lessons from mother to child. Hum Vaccin Immunother. (2017) 13:507–13. doi: 10.1080/21645515.2016.1244592, PMID: 27808600 PMC5360138

[13] Victor JR. Influence of maternal immunization with allergens on the thymic maturation of lymphocytes with regulatory potential in children: a broad field for further exploration. J Immunol Res. (2014) 2014:780386. doi: 10.1155/2014/780386, PMID: 25009823 PMC4070472

[14] Victor JR. Do different IgG repertoires play a role in B- and T-cell functional modulation during ontogeny? The "hooks without bait" theory. Immunol Cell Biol. (2020) 98:540–8. doi: 10.1111/imcb.12335, PMID: 32342552

[15] Futata E de Brito C Victor J Fusaro A Oliveira C Maciel M. Long-term anergy in orally tolerized mice is linked to decreased B7.2 expression on B cells. Immunobiology. (2006) 211:157–66. doi: 10.1016/j.imbio.2005.08.006, PMID: 16530083

[16] de Oliveira MG Lira AAL Sgnotto FDR Inoue AHS Beltrame GR da Silva D. Maternal immunization downregulates offspring TCD4 regulatory cells (Tregs) thymic maturation without implications for allergy inhibition. Scand J Immunol. (2018) 88:e12721. doi: 10.1111/sji.12721 30403024

[17] da Ressureicao Sgnotto F Garcia de Oliveira M de Lima Lira AA Bento-de-Souza L da Silva Duarte AJ Russo Victor J. Low doses of IgG from atopic individuals can modulate in vitro IFN-gamma production by human intra-thymic TCD4 and TCD8 cells: an IVIg comparative approach. Hum Vaccin Immunother. (2017) 13:1563–72. doi: 10.1080/21645515.2017.1299299, PMID: 28441069 PMC5512811

[18] Garcia de Oliveira M da Ressureição Sgnotto F Rodrigues de Sousa T Fagundes BO Duarte AJDS Victor JR. Preconceptional immunization with an allergen inhibits offspring thymic Th17 cells maturation without influence on Th1 and Th2 cells. Eur Cytokine Netw. (2020) 31:113–17. doi: 10.1684/ecn.2020.0452, PMID: 33270019

[19] Rodrigues de Sousa T da Ressureição Sgnotto F Oliveira Fagundes B Souza Santos L da Silva Duarte AJ Russo Victor J. IgG from atopic individuals can mediate non-atopic infant thymic and adult peripheral CD8 + TC2 skewing without influence on TC17 or TC22 cells. Eur. Ann. Allergy Clin Immunol. (2020) 53:161–7. doi: 10.23822/EurAnnACI.1764-1489.157 32548997

[20] Sgnotto FDR Oliveira MG Lira AAL Bento-de-Souza L Duarte AJDS Victor JR. Low doses of IgG from atopic individuals can modulate in vitro IFN-γ production by human intra-thymic TCD4 and TCD8 cells: an IVIg comparative approach. Hum Vaccin Immunother. (2017) 13:1563–72. doi: 10.1080/21645515.2017.1299299, PMID: 28441069 PMC5512811

[21] Sgnotto FDR de Oliveira MG Lira AAL Inoue AHS Titz TO Orfali RL. IgG from atopic dermatitis patients induces IL-17 and IL-10 production in infant intrathymic TCD4 and TCD8 cells. Int J Dermatol. (2018) 57:434–40. doi: 10.1111/ijd.13907, PMID: 29355930

[22] da Ressureição Sgnotto F Souza Santos L Rodrigues de Sousa T Feitosa de Lima J da Silva Oliveira LM Sanabani SS. IgG from HIV-1-exposed seronegative and HIV-1-infected subjects differently modulates IFN-γ production by Thymic T and B cells. J Acquir Immune Defic Syndr. (2019) 82:e56–60. doi: 10.1097/QAI.0000000000002182 31714433

[23] de Oliveira MG de Lima Lira AA da Ressureição Sgnotto F Inoue AHS Santos LS Nakamatsu BY. Maternal IgG impairs the maturation of offspring intrathymic IL-17-producing γδT cells: implications for murine and human allergies. Clin Exp Allergy. (2019) 49:1000–12. doi: 10.1111/cea.13393, PMID: 30929287

[24] Santos LS Sgnotto FDR Inoue AHS Padreca AF Menghini RP Duarte AJDS. IgG from non-atopic individuals induces in vitro IFN-γ and IL-10 production by human intra-thymic γδT cells: a comparison with atopic IgG and IVIg. Arch Immunol Ther Exp. (2019) 67:263–70. doi: 10.1007/s00005-019-00545-6, PMID: 31087106

[25] Inoue AHS Lira AAL De-Oliveira MG de Sousa TR Sgnotto FDR Duarte AJDS. The potential of IgG to induce murine and human Thymic maturation of IL-10+ B cells (B10) revealed in a pilot study. Cells. (2020) 9:2239. doi: 10.3390/cells9102239, PMID: 33027887 PMC7600151

[26] de Lima Lira AA De-Oliveira MG Inoue AHS Beltrame GR da Silva Duarte AJ Victor JR. Preconceptional allergen immunization can induce offspring IL-17 secreting B cells (B17): do they share similarities with regulatory B10 cells? Allergol Immunopathol. (2018) 46:454–9. doi: 10.1016/j.aller.2018.04.001 30082063

[27] de Oliveira MG Oliveira LM Lira AAL Sgnotto FDR Duarte AJDS Sato MN. Preconception allergen sensitization can induce B10 cells in offspring: a potential main role for maternal IgG. Allergy Asthma Clin Immunol. (2017) 13:22. doi: 10.1186/s13223-017-0195-8 28428801 PMC5392917

[28] Neco HVPD Teixeira VGDS da Trindade ACL Magalhães PMR de Lorena VMB Castellano LRC. Mediators go together: high production of CXCL9, CXCL10, IFN-γ, and TNF-α in HTLV-1-associated myelopathy/tropical spastic Paraparesis. AIDS Res Hum Retrovir. (2017) 33:1134–9. doi: 10.1089/aid.2016.0296, PMID: 28648091

[29] Starling AL Martins-Filho OA Lambertucci JR Labanca L de Souza Pereira SR Teixeira-Carvalho A. Gonçalves: Proviral load and the balance of serum cytokines in HTLV-1-asymptomatic infection and in HTLV-1-associated myelopathy/tropical spastic paraparesis (HAM/TSP). Acta Trop. (2013) 125:75–81. doi: 10.1016/j.actatropica.2012.09.012, PMID: 23022356

[30] Assone T Menezes SM de Toledo Gonçalves F Folgosi VA da Silva Prates G Dierckx T. Systemic cytokines and GlycA discriminate disease status and predict corticosteroid response in HTLV-1-associated neuroinflammation. J Neuroinflammation. (2022) 19:293. doi: 10.1186/s12974-022-02658-w 36482436 PMC9733207

[31] Kato M Imaizumi N Tanaka R Mizuguchi M Hayashi M Miyagi T. Elevation of the plasma levels of TNF receptor 2 in association with those of CD25, OX40, and IL-10 and HTLV-1 Proviral load in acute adult T-cell leukemia. Viruses. (2022) 14:751. doi: 10.3390/v14040751, PMID: 35458481 PMC9032861

[32] El Hajj H Hleihel R El Sabban M Bruneau J Zaatari G Cheminant M. Loss of interleukin-10 activates innate immunity to eradicate adult T-cell leukemia-initiating cells. Haematologica. (2021) 106:1443–56. doi: 10.3324/haematol.2020.264523, PMID: 33567810 PMC8094094

[33] Osame M Matsumoto M Usuku K Izumo S Ijichi N Amitani H. Chronic progressive myelopathy associated with elevated antibodies to human T-lymphotropic virus type I and adult T-cell leukemialike cells. Ann Neurol. (1987) 21:117–22. doi: 10.1002/ana.410210203, PMID: 2881513

[34] Muniz AL Rodrigues W Santos SB de Jesus AR Porto AF Castro N. Association of cytokines, neurological disability, and disease duration in HAM/TSP patients. Arq Neuropsiquiatr. (2006) 64:217–21. doi: 10.1590/s0004-282x2006000200009 16791359

[35] De Castro-Costa CM Araújo AQ Barreto MM Takayanagui OM Sohler MP da Silva EL. Proposal for diagnostic criteria of tropical spastic paraparesis/HTLV-I-associated myelopathy (TSP/HAM). AIDS Res Hum Retrovir. (2006) 22:931–5. doi: 10.1089/aid.2006.22.931, PMID: 17067261

[36] Lopez E Scott NE Wines BD Hogarth PM Wheatley AK Kent SJ. Low pH exposure during immunoglobulin G purification methods results in aggregates that avidly bind Fcγ receptors: implications for measuring fc dependent antibody functions. Front Immunol. (2019) 10:2415. doi: 10.3389/fimmu.2019.02415 31681303 PMC6797627

[37] Santos LS Sgnotto FDR Sousa TR Orfali RL Aoki V Duarte AJDS. IgG from atopic dermatitis patients induces non-atopic infant thymic invariant natural killer T (iNKT) cells to produce IL-4, IL-17, and IL-10. Int J Dermatol. (2019) 59:359–64. doi: 10.1111/ijd.14688 31631342

[38] Belkadi A Dietrich C Machavoine F Victor JR Leite-de-Moraes M. γδ T cells amplify Blomia tropicalis-induced allergic airway disease. Allergy. (2018) 74:395–8. doi: 10.1111/all.13618 30291622

[39] de Oliveira MG Oliveira LDM de Lima Lira AA Sgnotto FDR da Silva Duarte AJ Sato MN. Preconception allergen sensitization can induce B10 cells in offspring: a potential main role for maternal IgG. Allergy Asthma Clin Immunol. (2017) 13:22. doi: 10.1186/s13223-017-0195-8, PMID: 28428801 PMC5392917

[40] Tattermusch S Skinner JA Chaussabel D Banchereau J Berry MP McNab FW. Systems biology approaches reveal a specific interferon-inducible signature in HTLV-1 associated myelopathy. PLoS Pathog. (2012) 8:e1002480. doi: 10.1371/journal.ppat.1002480, PMID: 22291590 PMC3266939

[41] Matsuura E Kubota R Tanaka Y Takashima H Izumo S. Visualization of HTLV-1-specific cytotoxic T lymphocytes in the spinal cords of patients with HTLV-1-associated myelopathy/tropical spastic paraparesis. J Neuropathol Exp Neurol. (2015) 74:2–14. doi: 10.1097/NEN.0000000000000141, PMID: 25470342 PMC4336315

[42] Queiroz MAF Azevedo VN Amoras EDSG Moura TCF Guimarães Ishak MO Ishak R. +874A/T polymorphism among asymptomatic HTLV-1-infected individuals is potentially related to a worse prognosis. Front Microbiol. (2018) 9:795. doi: 10.3389/fmicb.2018.00795 29867783 PMC5968086

[43] Neco HVPC Teixeira VGS Trindade ACL Magalhães PMR Lorena VMB Vasconcelos LR. IL17A polymorphism is not associated with human T-Lymphotropic virus 1-associated myelopathy/tropical spastic Paraparesis. Viral Immunol. (2017) 30:298–301. doi: 10.1089/vim.2016.0152, PMID: 28410448

[44] Shafiei M Mozhgani SH. Th17/IL-17 Axis in HTLV-1-associated myelopathy tropical spastic Paraparesis and multiple sclerosis: novel insights into the immunity during HAMTSP. Mol Neurobiol. (2023) 60:3839–54. doi: 10.1007/s12035-023-03303-0, PMID: 36947318

[45] Uchiyama T Kamio M Kodaka T Tamori S Fukuhara S Amakawa R. Leukemic cells from some adult T-cell leukemia patients proliferate in response to interleukin-4. Blood. (1988) 72:1182–6. doi: 10.1182/blood.V72.4.1182.1182, PMID: 3262383

[46] Mori N Yamashita U Tanaka Y Nakata K Oda S Morimoto I. Interleukin-4 induces proliferation of adult T-cell leukemia cells. Eur J Haematol. (1993) 50:133–40. doi: 10.1111/j.1600-0609.1993.tb00081.x, PMID: 8472809

[47] Mori N Shirakawa F Murakami S Oda S Eto S. Characterization and regulation of interleukin-4 receptor in adult T-cell leukemia cells. Eur J Haematol. (1996) 56:241–7. doi: 10.1111/j.1600-0609.1996.tb01936.x, PMID: 8641393

[48] Domingos JA Soares LS Bandeira LM Bonin CM Vicente AC Zanella L. Cytokine profile and proviral load among Japanese immigrants and non-Japanese infected with HTLV-1 in a non-endemic area of Brazil. PLoS One. (2017) 12:e0174869. doi: 10.1371/journal.pone.0174869, PMID: 28376092 PMC5380323

[49] Brito-Melo GE Peruhype-Magalhães V Teixeira-Carvalho A Barbosa-Stancioli EF Carneiro-Proietti AB Catalan-Soares B. (GIPH): IL-10 produced by CD4+ and CD8+ T cells emerge as a putative immunoregulatory mechanism to counterbalance the monocyte-derived TNF-alpha and guarantee asymptomatic clinical status during chronic HTLV-I infection. Clin Exp Immunol. (2007) 147:35–44. doi: 10.1111/j.1365-2249.2006.03252.x, PMID: 17177961 PMC1810436

[50] Fujimoto T Nakamura T Furuya T Nakane S Shirabe S Kambara C. Relationship between the clinical efficacy of pentoxifylline treatment and elevation of serum T helper type 2 cytokine levels in patients with human T-lymphotropic virus type I-associated myelopathy. Intern Med. (1999) 38:717–21. doi: 10.2169/internalmedicine.38.717, PMID: 10480302

[51] Gessain A Mahieux R de Thé G. Genetic variability and molecular epidemiology of human and simian T cell leukemia/lymphoma virus type I. J Acquir Immune Defic Syndr Hum Retrovirol. (1996) 13:S132–45. doi: 10.1097/00042560-199600001-00022 8797716

[52] Schor D Porto LC Roma EH Castro-Alves J Villela AP Araújo AQC. Putative role of HLA polymorphism among a Brazilian HTLV-1-associated myelopathy/tropical spastic paraparesis (HAM/TSP) population. Sci Rep. (2023) 13:7659. doi: 10.1038/s41598-023-34757-w 37169817 PMC10173239

[53] Madureira MWS Queiroz MAF Lima SS Pereira LMS da Costa CA de Sousa MS. The FOXP3-924 a/G single nucleotide polymorphism may be associated with predictive factors for human T Lymphotropic virus 1 associated myelopathy. Viral Immunol. (2023) 36:136–43. doi: 10.1089/vim.2022.0149, PMID: 36745398

[54] Shimizu Y Yamanashi H Miyata J Takada M Noguchi Y Honda Y. VEGF polymorphism rs3025039 and human T-cell leukemia virus 1 (HTLV-1) infection among older Japanese individuals: a cross-sectional study. Bioengineering (Basel). (2022) 9:527. doi: 10.3390/bioengineering9100527, PMID: 36290496 PMC9598135

[55] Gomes YCP Silva MTT Leite ACCB Lima MASD Araújo AQC Silva Filho IL. Polymorphisms in HTLV-1 tax-responsive elements in HTLV-1-associated myelopathy/tropical spastic paraparesis patients are associated with reduced proviral load but not with disease progression. J Gen Virol. (2021) 102:1649. doi: 10.1099/jgv.0.001649, PMID: 34494950

[56] Aghamohammadi A Rafatpanah H Maghsoodlu M Tohidi N Mollahosseini F Shahabi M. Mannose binding lectin-associated serine protease 2 (MASP2) gene polymorphism and susceptibility to human T-lymphotropic virus type 1 (HTLV-1) infection in blood donors from Mashhad, Iran. Microbiol Immunol. (2022) 66:460–4. doi: 10.1111/1348-0421.13022, PMID: 35924689

[57] Levin MC Lee SM Kalume F Morcos Y Dohan FC Hasty KA. Autoimmunity due to molecular mimicry as a cause of neurological disease. Nat Med. (2002) 8:509–13. doi: 10.1038/nm0502-509, PMID: 11984596 PMC2703733

[58] Osame M. Pathological mechanisms of human T-cell lymphotropic virus type I-associated myelopathy (HAM/TSP). J Neurovirol. (2002) 8:359–64. doi: 10.1080/13550280260422668, PMID: 12402162

[59] Mozhgani SH Zarei-Ghobadi M Teymoori-Rad M Mokhtari-Azad T Mirzaie M Sheikhi M. Human T-lymphotropic virus 1 (HTLV-1) pathogenesis: a systems virology study. J Cell Biochem. (2018) 119:3968–79. doi: 10.1002/jcb.26546, PMID: 29227540

