# Supplementary figures and images for: Differential modulation of IL-4, IL-10, IL-17, and IFN-γ production mediated by IgG from Human T-lymphotropic virus-1 (HTLV-1) infected patients on healthy peripheral T (CD4+, CD8+, and γδ) and B cells
Source: Front Med (Lausanne). 2023 Aug 30;10:1239706. doi: 10.3389/fmed.2023.1239706 (PMC10498471; doi:10.3389/fmed.2023.1239706)

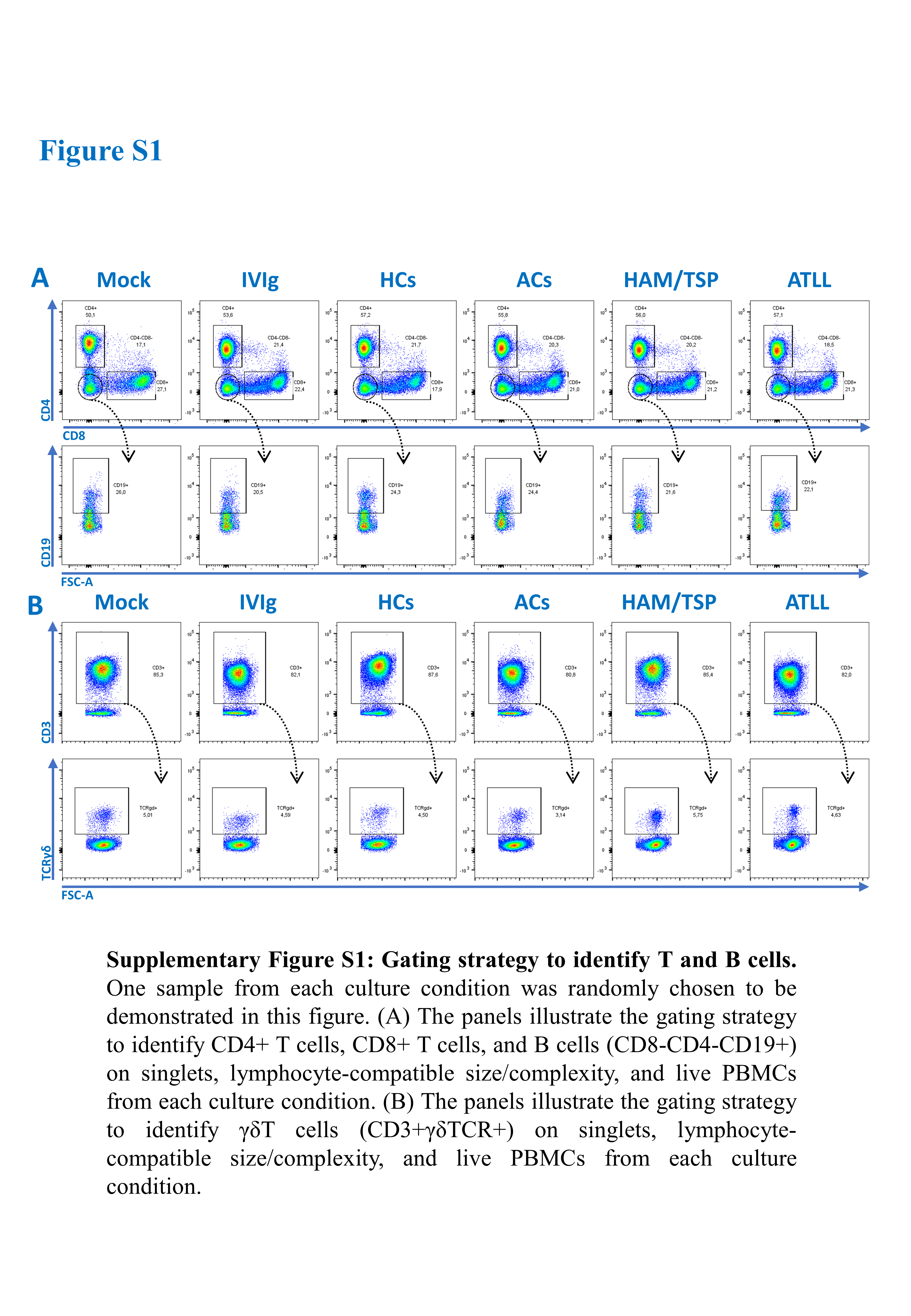

Supplement: Supplementary file 1 [file Image_1.TIF]

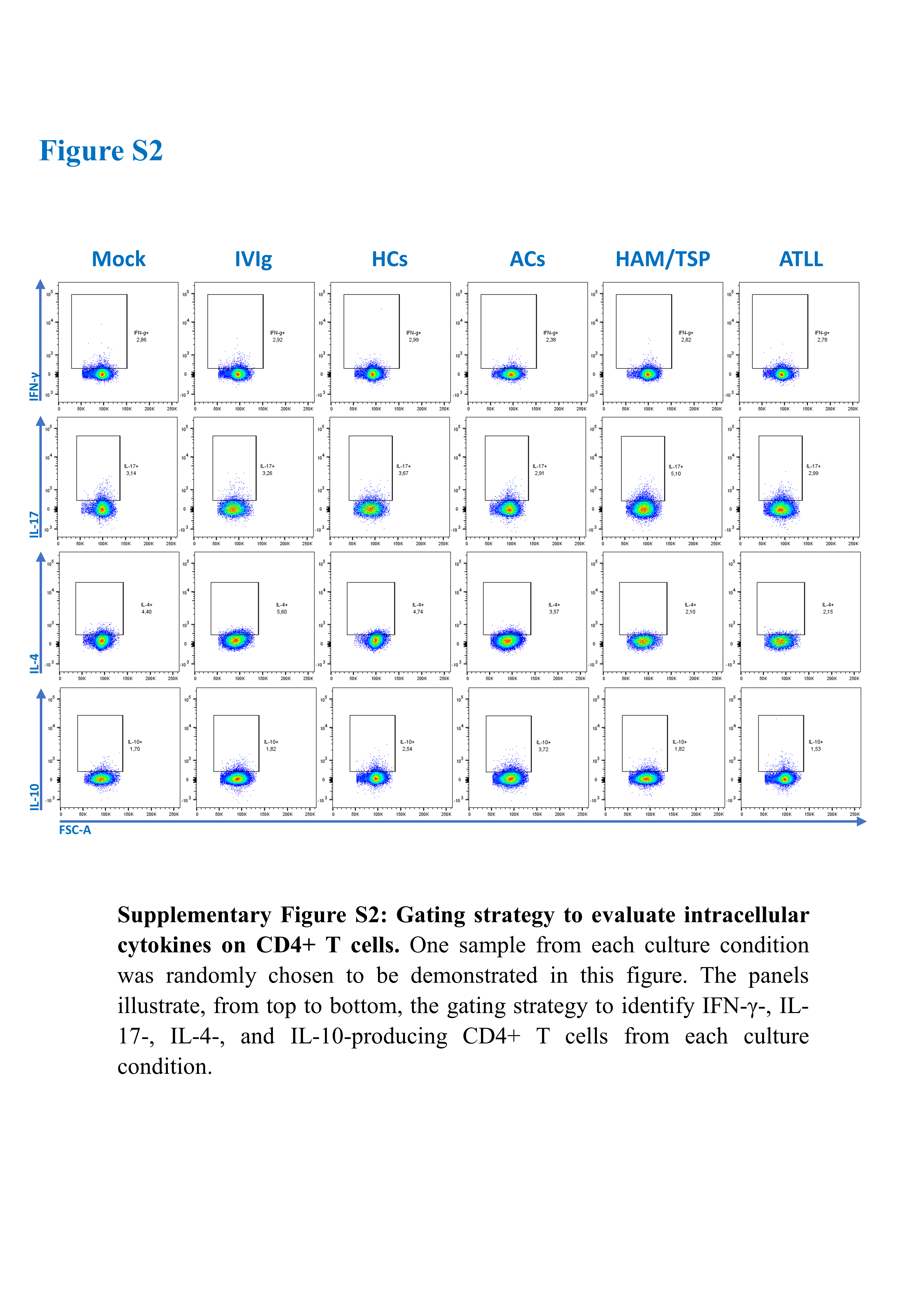

Supplement: Supplementary file 2 [file Image_2.TIF]

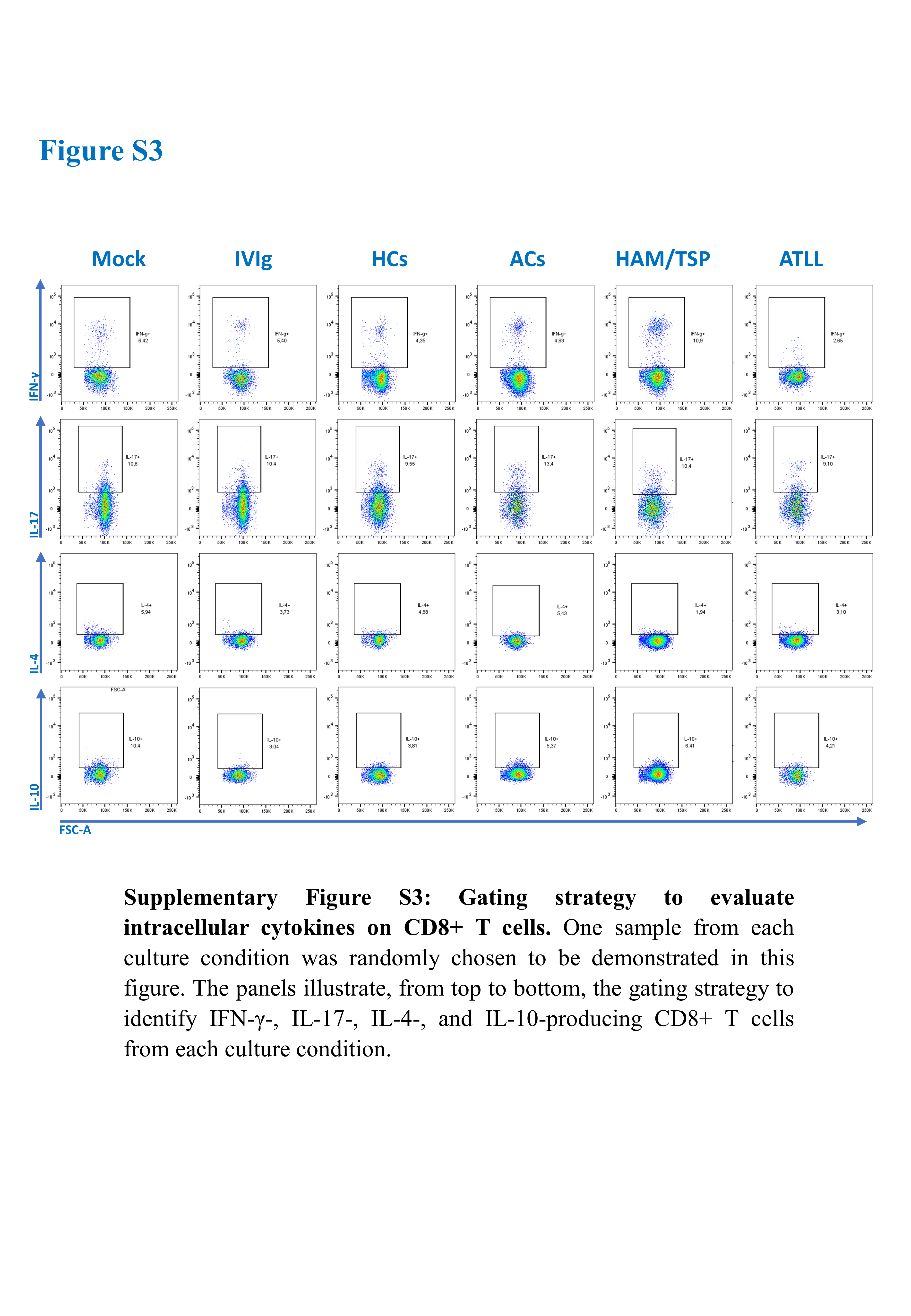

Supplement: Supplementary file 3 [file Image_3.TIF]

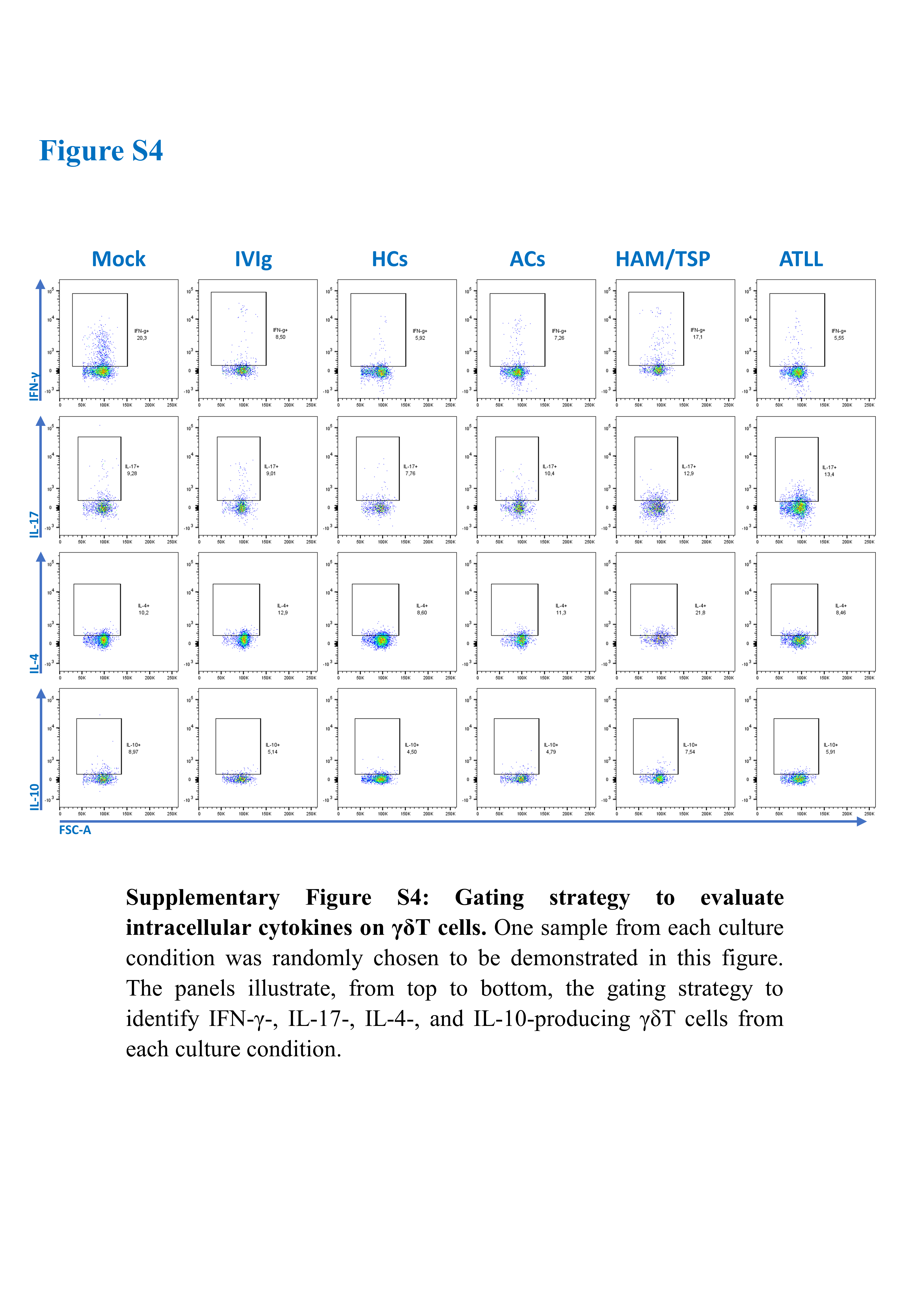

Supplement: Supplementary file 4 [file Image_4.TIF]

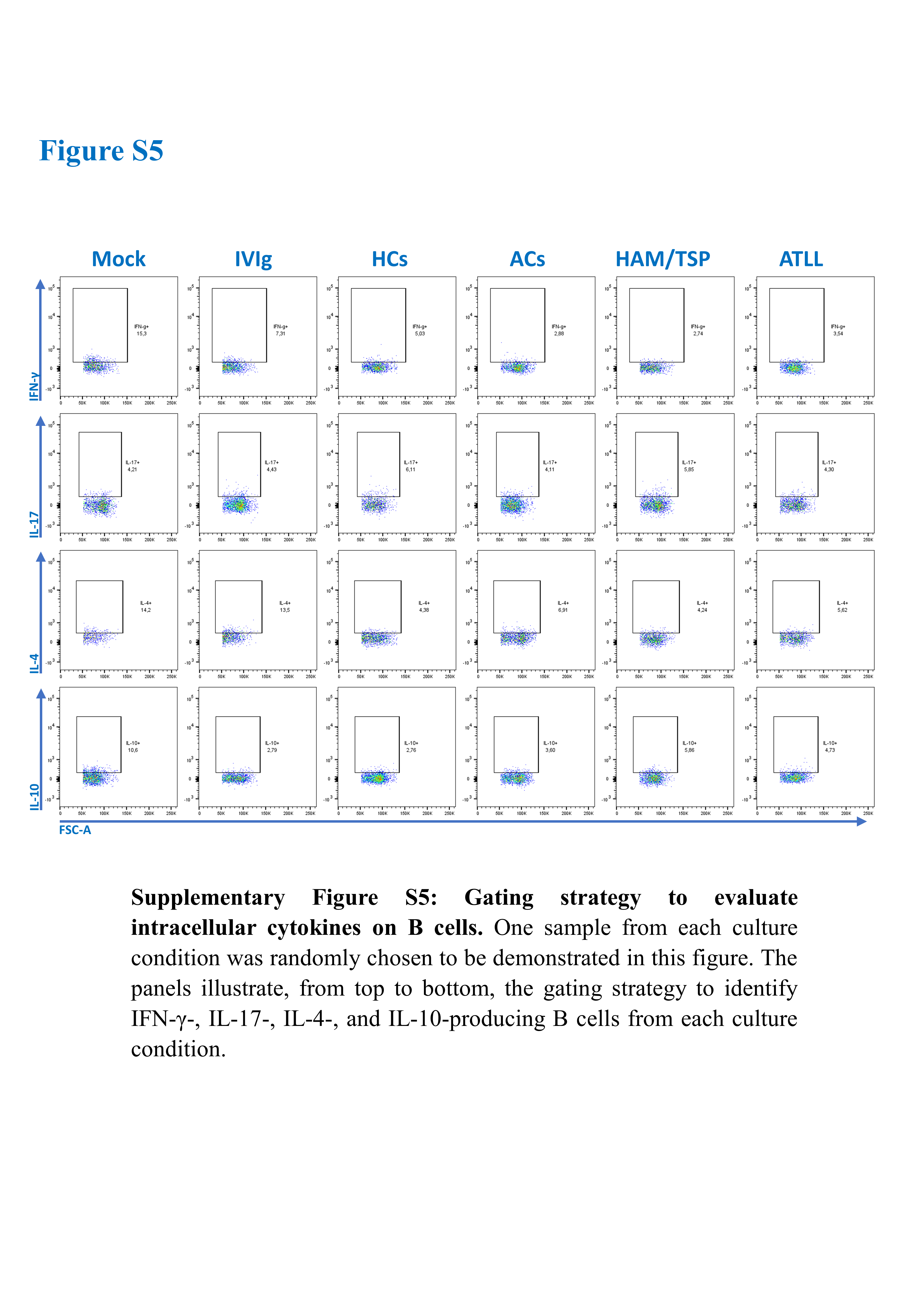

Supplement: Supplementary file 5 [file Image_5.TIF]
